# Upward comparison predicts an increase in state body dissatisfaction after fitspiration exposure

**DOI:** 10.1038/s41598-025-29830-5

**Published:** 2025-12-01

**Authors:** Gritt Ladwig, Kristine Schönhals, Julia A. Tanck, Hannah L. Quittkat, Silja Vocks

**Affiliations:** Department of Clinical Psychology and Psychotherapy, Institute of Psychology, Osnabrück University, Lise-Meitner-Str. 3, 49076 Osnabrück, Germany

**Keywords:** Eating disorders, Body dissatisfaction, Upward comparison, Social media, Fitspiration, Psychology, Human behaviour

## Abstract

As exposure to fitspiration images can lead to body dissatisfaction, it is important to understand which factors contribute to this effect. Therefore, this online experiment examined possible predictors of state body dissatisfaction following fitspiration exposure. In total, n = 131 women with and without self-reported eating disorder *(ED)* symptoms answered questionnaires on upward comparison*,* ED pathology, and drive for muscularity. Participants were then presented with 30 Instagram posts reflecting the fitspiration trend. Before and after fitspiration exposure, state body dissatisfaction was assessed. Results of a hierarchical regression analysis showed that when controlling for state body dissatisfaction before fitspiration exposure as well as participants’ age, upward comparison was the only significant predictor of state body dissatisfaction after fitspiration exposure. ED pathology and drive for muscularity did not predict an increase in state body dissatisfaction. Therefore, prevention programs that aim to mitigate harmful effects of fitspiration should focus on reducing upward comparison in women.

**Supplementary Information:**

The online version contains supplementary material available at 10.1038/s41598-025-29830-5.

## Introduction

*Fitspiration* is a trend on social media platforms like Instagram that putatively encourages women to improve their fitness and health^1^. Images reflecting this trend show thin and toned female bodies, often in sportswear^1^. Women have been found to evaluate a lean-muscular body type as especially attractive^2^, leading them to aspire to this athletic- or fit-looking body ideal^3^.

Despite its aim to promote a healthy lifestyle^1^, research has demonstrated a negative impact of fitspiration on body image, as evidenced by an increase in state body dissatisfaction after fitspiration exposure in female students^4^. Furthermore, research investigating this negative impact of fitspiration has revealed an important role of increased physical appearance comparisons after exposure to fitspiration, in turn leading to body dissatisfaction^5^.

An experimental study by Ladwig et al.^6^ specifically examined the relationship between fitspiration exposure and eating disorders (*ED*), and found that after exposure to fitspiration images, body dissatisfaction increased in women with self-reported EDs and women without mental disorders alike. However, a non-experimental study by Griffiths et al.^7^ revealed that more frequent exposure to fitspiration content was associated with increased symptom severity in individuals with self-reported EDs. Moreover, this association was mediated by physical appearance comparisons, with more frequent comparisons being related to greater ED symptom severity^7^. In a similar vein, Martin et al.^8^ reported a positive association between exposure to fitspiration content and higher levels of disordered eating impulses in female students. However, the direction of the effect remained unclear^8^. Investigating the direction of the association between fitspiration and ED pathology, a study by Christensen Pacella et al.^9^ found that female university students with ED symptoms who showed excessive exercise/muscle-building behaviors or higher cognitive restraint/purging were more likely to view fitspiration content in everyday life.

In light of these results, it appears that on the one hand, fitspiration content might have the potential to exacerbate ED symptoms, e.g., through increased comparisons^7^, and on the other hand, ED symptoms might make fitspiration exposure more likely^9^. However, it remains unclear whether ED pathology can predict the extent of state body dissatisfaction after fitspiration exposure.

Appearance pressure on social media, embodied by fitspiration content, can also lead to the internalization of a muscular body ideal^10^. The internalization of a lean and muscular body can in turn predict muscularity-oriented disordered eating^11^, which is associated with the drive for increased muscularity in women^12^. However, studies on the association of drive for muscularity with trait and state muscularity dissatisfaction in women are still lacking, even though the importance of muscularity in women is rising^2^. In the light of these results, beyond ED pathology and appearance comparisons, it appears crucial to investigate the role of drive for muscularity in the context of fitspiration exposure. A study by Holland and Tiggemann^13^ revealed higher levels of drive for muscularity, compulsive exercise, and disordered eating in women who actively post fitspiration content as compared to women who regularly post travel images. Yet, it remains unclear whether striving for greater muscularity can alter the effect of fitspiration exposure on state body dissatisfaction in women. In men, it has already been found that trait muscularity dissatisfaction appears to increase vulnerability to experiencing state muscularity dissatisfaction following fitspiration exposure. As it has been stated that muscularity dissatisfaction could constitute a facet of body dissatisfaction and both constructs are correlated in men^14^, it can cautiously be hypothesized that the drive for muscularity might influence the effect of fitspiration on state body dissatisfaction in women as well.

It is highly important to investigate possible predictors of state body dissatisfaction following fitspiration exposure, as identifying such risk factors that make women vulnerable to the effects of fitspiration content opens up the possibility of developing tailored prevention strategies. Hence, the present study investigated whether ED pathology, trait-like upward appearance comparisons, and drive for muscularity predict state body dissatisfaction following fitspiration exposure when controlling for state body dissatisfaction before fitspiration exposure as well as sociodemographic data (i.e., age). Participants’ age was included as body image changes across the lifespan^15,16^ and social media use differs between different age groups^17^. Our hypotheses were that the higher the scores on ED pathology, upward appearance comparisons, and drive for muscularity, the greater the state body dissatisfaction following fitspiration exposure.

## Method

### Participants

Participants were recruited via mailing lists, social media platforms, and messaging apps. This study analyzed a subsample of participants from a larger research project, part of which has been previously published^6^, resulting in a partial overlap of data. The analyzed subsample comprised n = 131 German-speaking women with and without current or past self-reported EDs, with a mean age of 28.44 years (SD = 7.42, range: 18—57) and a mean body mass index (BMI) of 23.64 kg/m^2^ (SD = 7.39, range: 14.67—58.59). Self-reported ED diagnoses were distributed as follows: current ED: n = 60 (anorexia nervosa (AN): n = 25, bulimia nervosa (BN): n = 15, binge eating disorder (BED): n = 11, other: n = 9), past ED: n = 18 (AN: n = 11, BN: n = 2, BED: n = 1, other: n = 4), no past or current ED: n = 53.

### Stimulus material

Fitspiration images were searched on Instagram using the hashtags #fitspiration and #fitspo. Images with similar hashtags, such as #fitnessmotivation, were still included if they matched the general image selection criteria, which included the following: The posts had to show a photo of a woman without large tattoos in different poses that were preferably not sexualized. We selected thirty posts showing women corresponding to the lean-muscular body ideal, with accompanying captions containing predominantly motivational quotes about sports and fitness. Informed consent was obtained from all content creators of the posts. As the number of possible images to be included in the study exceeded 30, two PhD students and four graduate students chose the 30 Instagram posts that corresponded most to the above-mentioned general image selection criteria^6^. Comments, likes, hashtags, and advertisements were removed from the posts, and captions exceeding the length of one sentence were translated into German.

### Measures

The *Upward Physical Appearance Comparison Scale (UPACS*^18^*,* German version^19^) assesses the extent to which respondents generally compare themselves with perceived more attractive persons on a scale ranging from 1 (*strongly disagree*) to 5 (*strongly agree*). The German UPACS^19^ has been assessed as being satisfactorily valid and reliable; acceptable internal consistency scores were found in women with EDs (McDonald’s ω_t_ = 0.93^19^). Internal consistency in the present study was excellent, at α = 0.93.

ED symptoms were measured using the *Eating Disorder Examination-Questionnaire (EDE-Q*^20^*,* German version^21^), which consists of the four subscales *Shape Concern* (eight items), *Weight Concern* (five items), *Eating Concern* (five items), *Restraint* (five items), and six further items measuring ED-related behaviors. The occurrence and frequency of ED symptoms are rated on a 7-point Likert scale ranging from 0 *(no days, not at all)* to 6 (*every day, markedly*), referring to the last 28 days. The EDE-Q has been evaluated as convergently valid and internally consistent in various samples including participants with EDs^22^. For the global score, internal consistency measured by Cronbach’s α was excellent, at α = 0.97.

The urge to increase one’s muscle was assessed using the subscale *Muscularity-Oriented Body Image* of the *Drive for Muscularity Scale (DMS*^23^*,* German version^24^). The subscale comprises seven items that were all rated on a scale ranging from 1 (*never*) to 6 (*always*). Psychometric properties of the DMS have been assessed in Brazilian women, revealing adequate internal consistency and test–retest reliability^25^. Cronbach’s α for this study was high, at α = 0.88.

State body dissatisfaction was measured using the *Body Image States Scale* (*BISS*^26^, German version^27^), which comprises six items rated on a scale ranging from 1 (*extremely satisfied, a great deal better*) to 9 (*extremely dissatisfied, a great deal worse*). The newly adapted German version (BISS-G^28^) has shown good to very good psychometric properties, including test-retest-reliability, convergent validity and internal consistency. In this study, internal consistency for the BISS pre-scores was excellent, at α = 0.94.

With regard to sociodemographic data, among other variables, participants were asked to indicate their age as well as their weight (in kilograms, kg) and height (in meters, m) in order to calculate their BMI (i.e., kg/m^2^).

### Procedure

Participants gained access to the survey (LimeSurvey version 5.3.21 + 220620) by clicking on a link. After participants provided informed consent, questionnaires on appearance comparisons (UPACS), ED symptoms (EDE-Q), and drive for muscularity (DMS) were collected at baseline. Throughout the survey, participants were only able to proceed if they had responded to all mandatory questions, ruling out missing data within the questionnaires. On the next working day, participants were sent a link to a survey where they were presented with 30 fitspiration Instagram posts, and were advised to view each image carefully for as long as they wished before clicking to see the next image. Before and after viewing all 30 posts, state body dissatisfaction (BISS) was measured. On average, participants responded to the second part of the survey after *M* = 2.45 days (*SD* = 2.37). At the end of the study, addresses and hotlines of help centers were provided to enable participants to seek support if viewing the fitspiration content led to feelings of unease. As reimbursement, participants received course credit or could be entered into a raffle to win vouchers. The study procedure was approved by the ethics committee of Osnabrück University. All methods were performed in accordance with the Declaration of Helsinki.

### Statistical analyses

Descriptive statistics and correlations between all variables (Pearson’s r) were computed using IBM SPSS (version 28.0.1.1). A hierarchical regression analysis was computed to predict the post-score on the *Body Image States Scale*, i.e., after viewing the fitspiration images. To control for state body dissatisfaction before viewing the fitspiration images, in Step 1, the pre-score on the *Body Image States Scale* was included as the sole predictor. Next, sociodemographic data, i.e., age (Step 2), the *Eating Disorder Examination-Questionnaire* global score (Step 3), the *Drive for Muscularity Scale* score (Step 4), and the *Upward Physical Appearance Comparison Scale* score (Step 5) were added successively as predictors. To determine the incremental value of each predictor, ΔR^2^ was computed and tested for significance in each step.

## Results

The *Body Image States Scale* pre-score (r = 0.91, p < 0.001), the *Eating Disorder Examination-Questionnaire* global score (r = 0.81, p < 0.001), the *Drive for Muscularity Scale* score (r = 0.25, p < 0.01), and the *Upward Physical Appearance Comparison Scale* score (r = 0.44, p < 0.001) showed significant positive correlations with the *Body Image States Scale* post-score. Participants’ age was the only examined predictor that did not correlate significantly with the *Body Image States Scale* post-score (r = 0.04, p = 0.62). All means and standard deviations as well as correlations between variables can be derived from Table 1.

**Table 1:** Means, standard deviations, and correlations of all variables included in the regression analysis.

Scale	*M*	*SD*	1	2	3	4	5	6
1. BISS_post	6.07	2.17	–					
2. BISS_pre	5.57	2.01	.91***	–				
3. Age	28.44	7.42	.04	.07	–			
4. EDE-Q	2.71	1.73	.81***	.86***	− .06	–		
5. DMS	2.57	1.09	.25**	.25**	− .12	.27***	–	
6. UPACS	3.55	0.96	.44***	.34***	− .31***	.49***	.28***	–

M = mean; SD = standard deviation; BISS_post = post-scores in the Body Image States Scale; BISS_pre = pre-scores in the Body Image States Scale; EDE-Q = Eating Disorder Examination-Questionnaire; DMS = Drive for Muscularity Scale; UPACS = Upward Physical Appearance Comparison Scale.

*p < .05. **p < .01. ***p < .001.

The results of the hierarchical regression analysis can be derived from Table 2. As expected, the *Body Image States Scale* pre-score significantly predicted the *Body Image States Scale* post-score, F(1,129) = 595.55, p < 0.001. The *Body Image States Scale* post-score prediction showed no incremental improvement when successively including age (Step 2), the *Eating Disorder Examination-Questionnaire* global score (Step 3), and the *Drive for Muscularity Scale* score (Step 4) into the model (all p > 0.05 for Δ*R*^2^). However, including the *Upward Physical Appearance Comparison Scale* score (Step 5) incrementally improved the prediction of the *Body Image States Scale* post-score, ΔF(1,125) = 14.54, p < 0.001. In Step 5, only the *Body Image States Scale* pre-score (β = 0.85) and the *Upward Physical Appearance Comparison Scale* score (β = 0.17) were significant predictors of the *Body Image States Scale* post-score (both p < 0.001).

**Table 2:** Hierarchical regression results for post-scores on the Body Image States Scale (BISS).

Variable	B	95% CI for B		SE B	β	R^2^	ΔR^2^
		*LL*	*UL*				
Step 1						.82	.82***
Constant	0.62**	0.16	1.09	0.24			
BISS_pre	0.98***	0.90	1.06	0.04	.91***		
Step 2						.82	.00
Constant	0.75	0.00	1.50	0.38			
BISS_pre	0.98***	0.90	1.06	0.04	.91***		
Age	− 0.01	− 0.03	0.02	0.01	− .02		
Step 3						.83	.00
Constant	0.84*	0.08	1.59	0.38			
BISS_pre	0.88***	0.72	1.03	0.08	.81***		
Age	0.00	− 0.02	0.02	0.01	.00		
EDE-Q	0.14	− 0.05	0.32	0.09	.11		
Step 4						.83	.00
Constant	0.76	− 0.08	1.61	0.43			
BISS_pre	0.87***	0.72	1.03	0.08	.81***		
Age	0.00	− 0.02	0.02	0.01	.00		
EDE-Q	0.14	− 0.05	0.32	0.09	.11		
DMS	0.03	− 0.12	0.19	0.08	.02		
Step 5						.84	.02***
Constant	− 0.66	− 1.75	0.43	0.55			
BISS_pre	0.91***	0.76	1.07	0.08	.85***		
Age	0.01	− 0.01	0.03	0.01	.04		
EDE-Q	0.01	− 0.18	0.19	0.10	.00		
DMS	− 0.02	− 0.16	0.13	0.08	− .01		
UPACS	0.38***	0.18	0.57	0.10	.17***		

CI = confidence interval; LL = lower limit; UL = upper limit; SE = standard error; BISS_pre = pre-scores on the Body Image States Scale; EDE-Q = Eating Disorder Examination-Questionnaire; DMS = Drive for Muscularity Scale; UPACS = Upward Physical Appearance Comparison Scale.

* p < .05. ** p < .01. *** p < .001.

## Discussion

The present study shows that trait-like upward appearance comparison predicts state body dissatisfaction after exposure to a lean-muscular body ideal in women with and without EDs when controlling for participant’s age, pre-existing state body dissatisfaction, ED symptoms and drive for muscularity. This finding appears to be in line with a study by Robinson et al.^29^, who identified appearance comparison as a predictor of increased state body dissatisfaction in women after exposure to images displaying a thin, athletic, or muscular ideal. However, in the study by Robinson et al.^29^, solely state appearance comparison predicted state body dissatisfaction, whereas trait appearance comparison did not moderate the effect of media exposure on state body dissatisfaction. This discrepant finding might be attributable to the type of measure employed, as Robinson et al.^29^ assessed general appearance comparison processes, while the present study specifically examined upward comparison. Future research could therefore include general appearance comparison as an additional predictor in order to differentiate between different types of comparison (i.e., upward vs. general appearance comparison). Overall, the present findings imply that appearance comparison is a risk factor that renders women who frequently engage in upward comparisons especially susceptible to the detrimental effects of fitspiration on body image.

Moreover, our finding that ED pathology did not predict state body dissatisfaction after fitspiration exposure corresponds to a study by Dignard and Jarry^30^, which showed reduced state body satisfaction after fitspiration exposure in women with high and low body appreciation alike. As body appreciation is inversely related with eating pathology^31^, it seems like women with high or low levels of ED symptoms react identically to fitspiration content (i.e., with increased state body dissatisfaction or decreased state body satisfaction, respectively), therefore, ED pathology has no predictive value. However, in contrast to the results by Dignard and Jarry^30^, a longitudinal study by Linardon^32^ revealed that in women, a more negative body image and higher levels of dietary restraint predicted higher rates of fitspiration comparisons, which in turn predicted higher negative body image at a later time point. With regard to these mixed results, one explanation for why ED symptoms did not predict state body dissatisfaction in the present study might be that the measures of ED pathology (i.e., *Eating Disorder Examination-Questionnaire*) and body dissatisfaction (i.e., *Body Image States Scale*) seem to partly assess the same construct, potentially causing a methodological issue. This assumption is underlined by the high correlation of the *Eating Disorder Examination-Questionnaire* and the *Body Image States Scale* scores (see Table 1). For example, the *Eating Disorder Examination-Questionnaire* contains the subscales *Weight Concern* and *Shape Concern*, which are related to measures of body dissatisfaction. Assuming that these two subscales have an overlap with the *Body Image States Scale*, solely the remaining two *Eating Disorder Examination-Questionnaire* subscales, namely *Restraint* and *Eating Concern*, would hold the potential to incrementally explain variance in state body dissatisfaction after fitspiration exposure, beyond state body dissatisfaction prior to exposure. Accordingly, this might lead to an underestimation of the impact of ED pathology. In order to investigate this hypothesis, we have performed an additional exploratory regression analysis, including the subscales *Restraint* and *Eating Concern*. Results revealed that neither subscale significantly predicted body dissatisfaction after fitspiration exposure (see Supplementary Materials). A further explanation for the lack of prediction by ED symptoms might lie in habituation effects: As demonstrated, for instance, by Christensen Pacella et al.^9^, women with EDs are more likely to be exposed to fitspiration content. Therefore, it seems plausible that women with EDs are accustomed to fitspiration images, which could neutralize the expected predictive role of ED pathology. This explanation might also apply to drive for muscularity, which likewise did not predict state body dissatisfaction after fitspiration exposure in the present study. To the best of our knowledge, no study has examined whether women who pursue a muscular body ideal are more likely to be exposed to fitspiration content, but it is conceivable that these women look for exercise recommendations on Instagram, again leading to habituation to fitspiration images. In turn, this could lead to women with a higher drive for muscularity being less sensitive to fitspiration content than was expected in the present study, rendering the extent of drive for muscularity unsuitable as a predictor of state body dissatisfaction after viewing fitspiration content.

### Limitations and clinical implications

Several limitations need to be considered when interpreting the present findings. First of all, as the study was a laboratory study with preselected fitspiration images, it seems likely that the setting did not reflect participants’ natural social media consumption behavior. To improve ecological validity, the duration of image exposure in this study was self-chosen by the participants. However, this in turn carries the risk of distortion effects, insofar as certain groups of participants, for example those with EDs, might have chosen a shorter or longer exposure time than others. As the image exposure duration was not recorded, possible effects of varying image viewing times could not be excluded and should therefore be investigated in future studies. Future research could also consider a standardized duration of image exposure to improve internal validity on the one hand, while additionally examining predictors of state body dissatisfaction in ecological momentary assessment studies to achieve high external validity on the other. Secondly, there was no attention check during image exposure. Even though participants had to actively click to see the next Instagram post, implying that they were focused on the images to a certain extent, future studies should include manipulation checks, for example, by asking questions on the image content (for example, see Rodgers et al.^33^). A third limitation of this study is that participants’ social media usage patterns were not investigated. Since higher social media use (i.e., more time spent on social media) can prospectively predict body dissatisfaction a year later^34^, social media usage patterns should be assessed and controlled for in future research. Fourth, to avoid priming effects, state measures were not assessed on the same day as the baseline trait-like measures (see Procedure). However, as it cannot be ruled out that the time lag between baseline measures and state measures might have influenced study results, future research should consider standardizing time intervals or assess both state and trait-like measures in the same day to find out if results hold. Fifth, participants were transparently informed that the aim of the study was to investigate the influence of Instagram posts on body image in women with and without eating disorders when entering the first part of the survey (baseline measures). This information was provided for ethical reasons, especially, to enable participants with ED symptoms to opt out before image exposure. However, even though in the second part of the study, i.e., right before image exposure, participants were only instructed to look at the following images carefully and intently, the participants’ knowledge of the study’s aim could have potentially biased their responses. Future studies should therefore consider masking the study purpose in order to rule out any possible prompting effects (for example, see Cohen et al.^35^).

Considering the clinical implications of this study, it appears crucial to reduce the potential harm caused by fitspiration images, as body dissatisfaction states an important risk factor for the development of eating disorders^36^. Specifically, it seems to be essential to develop prevention strategies for women who engage in upward comparisons. Such interventions addressing the reduction of upward comparison could promote media competence, since initial research has shown that media competence can reduce harmful influences of media^37^. Specifically, the inclusion of awareness-inducing video material that emphasizes the idealized nature of images in the media seems promising, as a study by Arendt et al.^38^ found that this type of psychoeducation can reduce unfavorable comparison processes. Moreover, media psychoeducation should include potential disadvantages of fitspiration exposure in general, as the trend not only seems to negatively impact body image^4^ but also appears to fail in its aim of engaging women in more frequent exercise^29^.

## Conclusion

Upward appearance comparison predicts state body dissatisfaction after fitspiration exposure when controlling for drive for muscularity and ED pathology. Therefore, prevention programs should address unfavorable comparison processes in women in order to buffer harmful effects of fitspiration.

## Supplementary Information

Below is the link to the electronic supplementary material.

Supplementary Material 1.

## Data Availability

The datasets generated during and analyzed during the current study are available from the corresponding author on reasonable request.

## References

[1] Tiggemann, M. & Zaccardo, M. “Strong is the new skinny”: A content analysis of #fitspiration images on Instagram. *J. Health Psychol.* **23**, 1003–1011. 10.1177/1359105316639436 (2018). 27611630 10.1177/1359105316639436

[2] Bozsik, F., Whisenhunt, B. L., Hudson, D. L., Bennett, B. & Lundgren, J. D. Thin is in? Think again: The rising importance of muscularity in the thin ideal female body. *Sex Roles* **79**, 609–615. 10.1007/s11199-017-0886-0 (2018).

[3] Cunningham, M. L. et al. The “not-so-healthy” appearance pursuit? Disentangling unique associations of female drive for toned muscularity with disordered eating and compulsive exercise. *Body Image* **42**, 276–286. 10.1016/j.bodyim.2022.06.002 (2022). 35841701 10.1016/j.bodyim.2022.06.002

[4] Prichard, I., Kavanagh, E., Mulgrew, K. E., Lim, M. S. C. & Tiggemann, M. The effect of Instagram #fitspiration images on young women’s mood, body image, and exercise behaviour. *Body Image* **33**, 1–6. 10.1016/j.bodyim.2020.02.002 (2020). 32062021 10.1016/j.bodyim.2020.02.002

[5] Jeronimo, F. & Carraca, E. V. Effects of fitspiration content on body image: A systematic review. *Eat. Weight Disord.* **27**, 3017–3035. 10.1007/s40519-022-01505-4 (2022). 36401082 10.1007/s40519-022-01505-4 PMC9676749

[6] Ladwig, G., Tanck, J. A., Quittkat, H. L. & Vocks, S. Risks and benefits of social media trends: The influence of “fitspiration”, “body positivity”, and text-based “body neutrality” on body dissatisfaction and affect in women with and without eating disorders. *Body Image* **50**, 101749. 10.1016/j.bodyim.2024.101749 (2024). 38850713 10.1016/j.bodyim.2024.101749

[7] Griffiths, S. et al. How does exposure to thinspiration and fitspiration relate to symptom severity among individuals with eating disorders? Evaluation of a proposed model. *Body Image* **27**, 187–195. 10.1016/j.bodyim.2018.10.002 (2018). 30359868 10.1016/j.bodyim.2018.10.002

[8] Martin, G., Portingale, J., Fuller-Tyszkiewicz, M. & Krug, I. Do appearance comparisons mediate the effects of thinspiration and fitspiration on body dissatisfaction, happiness, and disordered eating urges in women’s daily lives?. *Body Image* **46**, 108–116. 10.1016/j.bodyim.2023.05.006 (2023). 37271033 10.1016/j.bodyim.2023.05.006

[9] Christensen Pacella, K. A., Chen, Y., Forbush, K. T., Cushing, C. C. & Swinburne Romine, R. Prospectively predicting naturalistic exposure to fitspiration and thinspiration in young women with disordered eating by leveraging an ecological momentary assessment design. *Eat. Behav.* **50**, 101767. 10.1016/j.eatbeh.2023.101767 (2023). 37295375 10.1016/j.eatbeh.2023.101767 PMC10526890

[10] Roberts, S. R., Maheux, A. J., Hunt, R. A., Ladd, B. A. & Choukas-Bradley, S. Incorporating social media and muscular ideal internalization into the tripartite influence model of body image: Towards a modern understanding of adolescent girls’ body dissatisfaction. *Body Image* **41**, 239–247. 10.1016/j.bodyim.2022.03.002 (2022). 35306356 10.1016/j.bodyim.2022.03.002

[11] Louw, R., Donovan, C., Uhlmann, L., Ganson, K. T. & Piatkowski, T. Fit for whom? Gendered Impacts of the fit ideal on body image and behavior. *Sex Roles* **91**, 69. 10.1007/s11199-025-01622-1 (2025).

[12] Cheng, L. et al. Muscularity bias internalization moderates the associations of muscularity dissatisfaction with muscularity-oriented disordered eating and psychosocial well-being in men but not women. *Body Image* **51**, 101806 (2024). 39509919 10.1016/j.bodyim.2024.101806

[13] Holland, G. & Tiggemann, M. “Strong beats skinny every time”: Disordered eating and compulsive exercise in women who post fitspiration on Instagram. *Int. J. Eat Disord.* **50**, 76–79. 10.1002/eat.22559 (2017). 27302867 10.1002/eat.22559

[14] Valls, M., Bonvin, P. & Chabrol, H. Association between muscularity dissatisfaction and body dissatisfaction among normal-weight French men. *J. Men’s Health* **10**, 139–145. 10.1089/jomh.2013.0005 (2013).

[15] Quittkat, H. L., Hartmann, A. S., Düsing, R., Buhlmann, U. & Vocks, S. Body dissatisfaction, importance of appearance, and body appreciation in men and women over the lifespan. *Front. Psychiatry*10.3389/fpsyt.2019.00864 (2019). 31920737 10.3389/fpsyt.2019.00864 PMC6928134

[16] Hockey, A., Milojev, P., Sibley, C. G., Donovan, C. L. & Barlow, F. K. Body image across the adult lifespan: A longitudinal investigation of developmental and cohort effects. *Body Image* **39**, 114–124 (2021). 34271529 10.1016/j.bodyim.2021.06.007

[17] Faktenkontor. *Anteil der befragten Internetnutzer, die Instagram nutzen, nach Altersgruppen in Deutschland in den Jahren 2015 bis 2024.* https://de.statista.com/statistik/daten/studie/691584/umfrage/anteil-der-nutzer-von-instagram-nach-alter-in-deutschland/ (2024).

[18] O’Brien, K. S. et al. Upward and downward physical appearance comparisons: Development of scales and examination of predictive qualities. *Body Image* **6**, 201–206. 10.1016/j.bodyim.2009.03.003 (2009). 19447692 10.1016/j.bodyim.2009.03.003

[19] Schönhals, K. et al. Is my body better than yours? Validation of the German version of the upward and downward physical appearance comparison scales in individuals with and without eating disorders. *Front. Psychol.* **15**, 1390063. 10.3389/fpsyg.2024.1390063 (2024). 38899131 10.3389/fpsyg.2024.1390063 PMC11186468

[20] Fairburn, C. G. & Beglin, S. J. Assessment of eating disorders: Interview or self-report questionnaire?. *Int. J. Eat Disorder* **16**, 363–370 (1994). 7866415

[21] Hilbert, A., Tuschen-Caffier, B., Karwautz, A., Niederhofer, H. & Munsch, S. Eating Disorder Examination Questionnaire—German Version. *Diagnostica* (2016).

[22] Hilbert, A., Tuschen-Caffier, B., Karwautz, A., Niederhofer, H. & Munsch, S. Eating disorder examination-questionnaire. *Diagnostica* **53**, 144–154. 10.1026/0012-1924.53.3.144 (2007).

[23] McCreary, D. R. & Sasse, D. K. An exploration of the drive for muscularity in adolescent boys and girls. *J. Am. Coll. Health* **48**, 297–304. 10.1080/07448480009596271 (2000). 10863873 10.1080/07448480009596271

[24] Waldorf, M., Cordes, M., Vocks, S. & McCreary, D. “Ich wünschte, ich wäre muskulöser”: Eine teststatistische Überprüfung der deutschsprachigen Fassung der drive for muscularity scale (DMS). *Diagnostica*10.1026/0012-1924/a000106 (2014).

[25] de Carvalho, P. H. B., Oliveira, F. d. C., Neves, C. M., Meireles, J. F. F. & Ferreira, M. E. C. Is the Drive for Muscularity Scale a valid and reliable instrument for young adult women? *Body Image* **29**, 1–5 (2019). 10.1016/j.bodyim.2019.02.001 10.1016/j.bodyim.2019.02.001 30763801

[26] Cash, T. F., Fleming, E. C., Alindogan, J., Steadman, L. & Whitehead, A. Beyond body image as a trait: The development and validation of the body image states scale. *Eat. Disord.* **10**, 103–113. 10.1080/10640260290081678 (2002). 16864251 10.1080/10640260290081678

[27] Vocks, S., Legenbauer, T. & Heil, A. Food intake affects state body image: Impact of restrained eating patterns and concerns about eating, weight and shape. *Appetite* **49**, 467–475. 10.1016/j.appet.2007.03.006 (2007). 17459521 10.1016/j.appet.2007.03.006

[28] Vogel, T., Schönhals, K. & Vocks, S. Das Körperbild als Momentaufnahme - Validierung der deutschsprachigen Body Image States Scale (BISS). *Gemeinsamer Kongress der Deutschen-Adipositas Gesellschaft (DAG) und der Deutschen Gesellschaft für Essstörungen (DGESS)*, (Stuttgart, 2025).

[29] Robinson, L. et al. Idealised media images: The effect of fitspiration imagery on body satisfaction and exercise behaviour. *Body Image* **22**, 65–71. 10.1016/j.bodyim.2017.06.001 (2017). 28654826 10.1016/j.bodyim.2017.06.001

[30] Dignard, N. A. L. & Jarry, J. L. The, “little red riding hood effect:” Fitspiration is just as bad as thinspiration for women’s body satisfaction. *Body Image* **36**, 201–213. 10.1016/j.bodyim.2020.11.012 (2021). 33360477 10.1016/j.bodyim.2020.11.012

[31] Linardon, J., McClure, Z., Tylka, T. L. & Fuller-Tyszkiewicz, M. Body appreciation and its psychological correlates: A systematic review and meta-analysis. *Body Image* **42**, 287–296 (2022). 35878528 10.1016/j.bodyim.2022.07.003

[32] Linardon, J. Investigating longitudinal bidirectional associations between appearance comparisons to fitspiration content on Instagram, positive and negative body image, and dietary restraint. *Eat. Disord* **31**, 450–463. 10.1080/10640266.2023.2190973 (2023). 36935581 10.1080/10640266.2023.2190973

[33] Rodgers, R. F., Paxton, S. J. & Wertheim, E. H. Do images speak louder than words? Effects of body positive and fitspiration quotes and images on state body image in women and men. *Sex Roles* **91**, 10. 10.1007/s11199-024-01553-3 (2024).

[34] Marques, M. D., Paxton, S. J., McLean, S. A., Jarman, H. K. & Sibley, C. G. A prospective examination of relationships between social media use and body dissatisfaction in a representative sample of adults. *Body Image* **40**, 1–11 (2022). 10.1016/j.bodyim.2021.10.008 10.1016/j.bodyim.2021.10.008 34768094

[35] Cohen, R., Fardouly, J., Newton-John, T. & Slater, A. #BoPo on Instagram: An experimental investigation of the effects of viewing body positive content on young women’s mood and body image. *New Media Soc.* **21**, 1546–1564. 10.1177/1461444819826530 (2019).

[36] Stice, E., Gau, J. M., Rohde, P. & Shaw, H. Risk factors that predict future onset of each DSM-5 eating disorder: Predictive specificity in high-risk adolescent females. *J. Abnorm. Psychol.* **126**, 38–51. 10.1037/abn0000219 (2017). 27709979 10.1037/abn0000219 PMC5215960

[37] Tobin, L. N., Sears, C. R. & von Ranson, K. M. Two eating disorder preventive interventions reduce attentional biases in body-dissatisfied university women: A cluster randomized controlled trial. *J. Consult. Clin. Psychol.* **90**, 911–924. 10.1037/ccp0000768 (2022). 36395030 10.1037/ccp0000768

[38] Arendt, F., Peter, C. & Beck, J. Idealized female beauty, social comparisons, and awareness intervention material evidence for preventive effects in young women. *J. Med. Psychol. Ger.* **29**, 188–197. 10.1027/1864-1105/a000181 (2017).

