# Supplementary material for: Upward comparison predicts an increase in state body dissatisfaction after fitspiration exposure
Source: Sci Rep. 2025 Dec 1;15:42936. doi: 10.1038/s41598-025-29830-5 (PMC12673077; doi:10.1038/s41598-025-29830-5)
Supplement: Supplementary file 1 — Supplementary Material 1. [file 41598_2025_29830_MOESM1_ESM.pdf]

Supplementary Material: Regression Analysis with the subscales

Supplement Table 1

Exploratory hierarchical Regression results for post-scores in the Body Image States Scale (BISS) with the two subscales "Restraint" and "Eating Concern" of the Eating Disorder Examination-Questionnaire (EDE-Q).

Variable	*B*	95% CI for *B*		*SE B*	β	R^2	ΔR^2
		LL	*UL*				
Step 1						.82	.82***
Constant	0.62**	0.16	1.09	0.24			
BISS_pre	0.98***	0.90	1.06	0.04	.91***		
Step 2						.82	.00
Constant	0.75	0.00	1.50	0.38			
BISS_pre	0.98***	0.90	1.06	0.04	.91***		
Age	-0.01	-0.03	0.02	0.01	-.02		
Step 3						.82	.00
Constant	0.75	-0.02	1.52	0.39			
BISS_pre	0.93***	0.79	1.06	0.07	.86***		
Age	0.00	-0.02	0.02	0.01	.00		
EDE-Q_r	0.05	-0.09	0.18	0.07	.04		
EDE_Q_e	0.03	-0.14	0.20	0.09	.03		
Step 4						.82	.00
Constant	0.67	-0.18	1.53	0.43			
BISS_pre	0.92***	0.79	1.06	0.07	.86***		
Age	0.00	-0.02	0.02	0.01	.00		
EDE-Q_r	0.05	-0.09	0.18	0.07	.04		
EDE-Q_e	0.03	-0.14	0.20	0.09	.03		
DMS	0.03	-0.12	0.19	0.08	.02		
Step 5						.84	.02***
Constant	-0.67	-1.72	0.38	0.53			
BISS_pre	0.92***	0.79	1.05	0.07	.85***		
Age	0.01	-0.01	0.03	0.01	.04		
EDE-Q_r	-0.01	-0.14	0.12	0.07	-.01		
EDE-Q_e	0.01	-0.16	0.17	0.08	.01		
DMS	-0.02	-0.16	0.13	0.08	-.01		
UPACS	0.38***	0.19	0.57	0.10	.17***		

Note. CI = confidence interval; *LL* = lower limit; *UL* = upper limit; BISS_pre = pre-scores in the Body Image States Scale; EDE-Q_r = Eating Disorder Examination-Questionnaire, Restraint Scale; EDE-Q_e = Eating Disorder Examination-Questionnaire, Eating Concern Subscale; DMS = Drive for Muscularity Scale; UPACS = Upward Physical Appearance Comparison Scale.

* $p < .05$. ** $p < .01$. *** $p < .001$.
